# From hospital dentistry to health systems: advancing special care dentistry through education, integration, and technology

**DOI:** 10.3389/froh.2026.1910418

**Published:** 2026-07-17

**Authors:** Sujay A. J. Mehta, Martha Mutis, David Kurtzman, Eric C. Sung

**Affiliations:** 1 Vancouver Orofacial Pain, Vancouver, BC, Canada; 2 Department of Medicine, Faculty of Health Sciences, McMaster University, Hamilton, ON, Canada; 3 Special Care Dentistry Association, New York, NY, United States; 4 American Association of Hospital Dentistry, Augusta, GA, United States; 5 Section of Special Patient Care and Maxillofacial Prosthetics, UCLA School of Dentistry, Los Angeles, CA, United States

**Keywords:** competency based education, health systems integration, hospital dentistry, interprofessional education, oral health equity, patient safety, special care dentistry

## Abstract

Special Care Dentistry has been defined by the populations it serves rather than by standardized frameworks, clinical pathways, and accountable care models. This population-centered framing has left the field dependent on the goodwill of individual clinicians rather than on reproducible standards. The results are wide variability in training, inconsistent care delivery, and persistent exclusion of medically complex, disabled, and frail patients from oral health systems not designed to serve them. We argue that this absence of systematization is a patient safety problem, and hospital dentistry offers a practical framework for addressing it that the field has not fully engaged. Hospital-based dental practice has developed the interprofessional competencies, documentation standards, and referral protocols that SCD requires but has not yet codified. The American Association of Hospital Dentistry holds the institutional experience to serve as an intellectual steward for the field's transformation. Our central hypothesis is that embedding SCD within the educational infrastructure of hospital dentistry, and aligning it with competency-based, value-driven, and interprofessional frameworks, represents a testable pathway toward more coherent and equitable care for populations with complex needs. We draw on evidence across workforce development, care delivery, and health policy to identify gaps in current systems and proof of concept from hospital-based models. The stakes are clear: without a systems-level response, SCD will continue to function as an accommodation rather than a discipline, and the populations it is meant to serve will continue to bear the consequences.

## Introduction

Health systems worldwide are confronting a growing convergence of aging populations, rising prevalence of disability, chronic disease, and medical complexity, alongside persistent inequities in access to oral health care. Individuals with disabilities, complex medical conditions, and frailty experience a disproportionate burden of oral disease, unmet need, and avoidable complications. That burden is not distributed randomly. A 2024 national spatial analysis found that nearly 1.7 million Americans lack access to a dental clinic within a 30-minute drive, with rural areas, communities with higher levels of poverty, and communities experiencing racial segregation disproportionately represented among dental care shortage areas (1). Emergency department data reinforce this picture: a 2026 study confirmed that socioeconomic disadvantage is independently associated with avoidable dental emergency department visits, with 79 percent of non-traumatic dental ED presentations considered preventable, a figure that reflects structural failure rather than individual behavior (2). Geographic distance compounds this inequity further, particularly for populations with mobility limitations and transportation barriers (3).

The dominant model of oral health delivery, fee-for-service private practice, is structurally misaligned with the needs of these populations. While many with low income go without any dental care at all, those who can pay are subjected to procedure-driven care disconnected from the holistic, patient-centered, and medically integrated care (4). This two-tier dynamic is a product of how private practice dentistry is organized, not an incidental feature of it. Medically complex, disabled, and frail patients are precisely those whose care demands exceed what conventional dental workflows accommodate, and they are systematically excluded from the population that private practice serves.

Oral health systems remain poorly equipped to respond to this complexity in a coordinated and sustainable manner. Within dentistry, these challenges have historically been addressed under the broad umbrella of Special Care Dentistry (SCD), a field defined more by the populations it serves than by standardized educational frameworks, clinical pathways, or system-level integration. Gallagher and Fiske's foundational analysis of SCD noted that established clinicians in the field work “across primary and hospital settings,” coordinating with colleagues across health, social services, and the voluntary sector, a description of integrated care that most of the profession has yet to operationalize at scale (5). Older adults alone represent a rapidly growing population whose oral health needs intersect extensively with systemic disease, polypharmacy, and functional decline, requiring coordinated interdisciplinary strategies that extend well beyond what the conventional dental encounter can provide (6).

Despite its essential role, SCD has functioned primarily as an accommodation service rather than a systematized clinical discipline, a categorization reflected in its absence from most national workforce strategies. This marginalization has contributed to wide variability in training, inconsistent models of care delivery, and fragmented policy recognition. Hospital dentistry, however, has long operated at the intersection of medicine, dentistry, and public health, providing care to patients with complex medical needs, disabilities, and acute conditions within integrated clinical environments. Hospital dentistry offers a pragmatic and scalable foundation for advancing SCD from a collection of individualized practices to a systematized discipline aligned with contemporary standards of quality, safety, and equity.

## Purpose of this perspective

We argue that hospital dentistry, long operating at the intersection of oral health and medical complexity, offers an underutilized but scalable framework for systematizing SCD, and that absence of such systematization constitutes a patient safety problem, not merely a professional identity problem. This article advances a systems-oriented agenda for SCD that emphasizes structured education and competency development, standardized clinical pathways, and the responsible use of technological innovation to support care for individuals with complex needs.

The absence of systematization across education, clinical practice, and policy in SCD represents a workforce and access challenge, as well as a patient safety and quality concern. By reframing SCD as an essential function of integrated health systems rather than a niche domain within dentistry, this perspective seeks to provide an intellectual foundation for the Research Topic “Transforming Special Care Dentistry: Frameworks for Education, Clinical Excellence, and Technological Innovation.”

Our aims are to catalyze interdisciplinary dialogue, inform future research, and support the development of sustainable models of care that address people with disabilities, chronic conditions, and age-related frailty across diverse health system contexts.

## From hospital dentistry to health systems: reframing special care dentistry

Health systems increasingly demand standardized approaches to managing complexity in patient care, with attention to patient safety, accountability, and coordination across disciplines. Hospital dentistry offers a distinct and under-recognized framework to position SCD as a health system function rather than a peripheral clinical niche. In hospital settings, medical complexity is the norm rather than the exception. Patients receiving care in these environments frequently present with multimorbidity, polypharmacy, cognitive or physical disability, and heightened procedural risk, conditions that necessitate close interprofessional collaboration with physicians, nurses, pharmacists, and allied health professionals. These conditions mirror the core clinical realities and responsibilities of SCD and highlight the alignment between hospital dentistry and modern models of integrated care.

Hospital dentistry incorporates principles foundational to modern health but inconsistently applied within dentistry as a whole. These include structured risk assessment, documentation standards aligned with medical records, interprofessional communication, shared decision-making, and explicit attention to patient safety and quality improvement. Hospital-based models necessitate explicit protocols and accountability mechanisms to manage complexity at scale, the kind of systems-level organization that can advance SCD beyond individualized accommodations toward standardized and reproducible models of care.

The experience of the Special Care Dentistry Association (SCDA), and to a larger extent, American Association of Hospital Dentistry (AAHD) reflects this trajectory. SCDA members have provided oral health care in diverse settings and integrated care delivery models, addressing the needs of patients who cannot safely or effectively be managed in the conventional private practice settings. SCDA have, by necessity, developed the workflows, documentation standards, and referral protocols that SCD advocates call for. These are clinical priorities shaped by patient populations whose needs demand them, not theoretical constructs. The question of whether these contributions have been systematically translated into formal education standards, policy frameworks, or broader professional recognition within dentistry remains important.

Community-based disability care, geriatric dentistry, and long-term care share core clinical and organizational challenges with hospital-based practice. Hospital dentistry offers a unifying educational, training and workforce framework for these diverse SCD settings, built around shared competencies, clinical pathways and quality expectations. By focusing attention on how complexity is managed rather than on which diagnostic categories are served, this approach encourages integrating SCD into health systems that support equity, safety, and sustainability. The growing recognition of oral-systemic connections, including bidirectional relationships between oral health and cardiac, metabolic, and renal conditions, adds weight to this argument (7). The periodontal-cerebrovascular relationship is instructive: a 2025 systematic review and meta-analysis of 36 studies encompassing more than 7.6 million participants confirmed that periodontal disease is an independent risk factor for stroke, with individuals with periodontitis carrying 72 percent higher odds of stroke compared to periodontally healthy individuals (8). A contemporaneous review published in the flagship journal of the American Stroke Association mapped the inflammatory pathways linking periodontal bacteremia to cerebrovascular injury, further reinforcing the case that hospital-based oral health care is a medically necessary component of complex patient management rather than a supplementary service (9). Taken together, these findings support repositioning SCD from a fragmented collection of practices into a more coherent discipline capable of meeting current and future population health needs.

## The role of the American association of hospital dentistry in advancing special care dentistry

Hospital dentistry plays a central but often under-acknowledged role in the oral health care needs of medically complex and vulnerable patients either unable to access, or inappropriate to treat within, conventional private practice settings. AAHD was established in 1937 to support dentists practicing in hospital and institutional settings where oral health is inseparable from broader medical, ethical and systemic considerations. From this perspective, AAHD has long contributed to the practical advancement of SCD, even in the absence of formal specialty recognition or standardization frameworks within the dental profession.

Hospital-based dental practice has historically served patients with complex medical presentations, disabilities, and acute or chronic health needs who cannot safely be treated in conventional private practice, including patients for whom treatment by dentists without formal residency training would carry unacceptable clinical risk. AAHD members have developed expertise in navigating medical comorbidity, coordinating care across disciplines, and delivering oral health services within environments governed by institutional protocols, patient safety standards, and regulatory oversight. These experiences position hospital dentistry as a formative training environment for SCD, reinforcing competencies beyond clinical technique to include risk assessment, interprofessional communication and systems-based practice.

Beyond clinical care, AAHD has served as a professional home for knowledge exchange, mentorship, and advocacy related to the care of medically complex populations. Through educational programming, professional networking, and engagement with broader health care conversations, AAHD has helped sustain and disseminate practices aligned with SCD principles. The contributions of hospital dentistry have not always been recognized within prevailing definitions of SCD, which have tended to emphasize patient categories rather than care environments or system functions.

AAHD operates within a broader ecosystem of Special Care Dentistry organizations, including those focused on disability-specific care and geriatric oral health. Hospital dentistry offers a unifying systems perspective that can bridge these domains, providing a framework for standardizing competencies, aligning care pathways, and integrating oral health into multidisciplinary health systems. AAHD's experience demonstrates how SCD can be operationalized within complex care environments, complimenting and strengthening the work of related organizations.

As SCDA seeks greater coherence, recognition, and impact, AAHD's role is not to define the field in a narrow manner, but to contribute a systems-oriented foundation informed by decades of hospital-based practice. Articulating how hospital dentistry has already embodied many of the educational, clinical, and collaborative principles central to SCD, AAHD can help guide the field toward more consistent standards, stronger integration with health systems, and sustainable models of care.

## Defining the systematization gap in special care dentistry

The gap between what SCD aspires to accomplish and what current systems actually deliver is visible across education and competency frameworks, clinical pathways and standards of care, and policy, recognition, and workforce structures. Each domain reinforces the others; progress in any one requires attention to all.

## Education and training: from exposure to competency-based special care dentistry practice

Education is the most under-appreciated yet most critical domain for closing the systems gap in SCD. Despite growing calls for integrating oral health into medicine, the rising prevalence of chronic disease and disability, an aging population, and the acknowledged limits of the 4-year dental curriculum, dental education has largely relied on exposure-based approaches to prepare clinicians for SCD practice. Exposure to diverse patient populations is necessary, but insufficient to prepare dental graduates without formal residency programs to manage complexity, coordinate care, communicate across health professions, and navigate clinical risk within integrated health systems.

A 2024 scoping review confirmed that dental students frequently graduate without the foundational knowledge and clinical experience required to manage geriatric, medically compromised, and complex special needs populations, and that this clinical gap has persisted across multiple review cycles internationally (10). The pre-doctoral dental curriculum is genuinely challenged to address SCD competencies in depth. The 4-year program must balance foundational biomedical knowledge, clinical skill development, communication, ethics, and interprofessional collaboration. While SCD principles are incorporated at dental schools to varying degrees, the curriculum tends to frame SCD as a peripheral adjunct rather than a core domain requiring distinct competencies and longitudinal skill development.

Post-graduate training in Advanced Dental Education such as General Practice Residency (GPR) and Advanced Education in General Dentistry (AEGD) programs, offers a compelling model for moving from exposure to competency-based SCD education. They involve comprehensive and interdisciplinary care as part of their core standard. In these programs, medical complexity is routine rather than episodic, which demands that trainees engage in structured risk assessments, interdisciplinary communication, and shared decision-making as part of everyday practice. A national survey of U.S. GPR and AEGD program directors found that while most programs teach residents to identify common medical conditions including diabetes and hypertension, significant curricular gaps persist for obesity management and other complex systemic conditions, and the degree of primary care integration into postdoctoral dental training varies considerably across programs (11). A separate evaluation of AEGD and Advanced Education in Pediatric Dentistry (AEPD) residents at rural community health centers found that self-reported preparedness to treat patients with complex healthcare needs remained a development area even after postdoctoral clinical rotation, suggesting that structured frameworks rather than rotational exposure alone are needed (12).

These training gaps are not new. Program directors of GPR and AEGD programs identified medically complex patient care as a critical and underfunded curricular priority more than two decades ago, flagging inadequate training in special patient care alongside chronic concerns about resident stipends, faculty shortages, and the perceived devaluation of hospital-based programs by institutional administrators (13). That these structural tensions persist in current literature suggests they are problems of systemic undervaluation within health system funding frameworks, not simply resource allocation. National Dental Matching Program data reinforce this picture: in the 2024 Match cycle, 196 of 710 U.S. GPR positions went unfilled, and AEGD programs left 111 of 301 positions vacant, even as the overall applicant pool continued a sustained decline from a 2021 peak (14).

The simultaneous contraction of both supply and demand points to structural barriers beyond debt load and stipend inadequacy. New graduates increasingly enter private practice and corporate dental settings that offer immediate salaries substantially higher than residency stipends, making the financial case for an additional year of training difficult to sustain against competing economic pressures (13, 14). Fewer trainees entering AEGD and GPRs means fewer clinicians prepared to serve medically complex populations. This trend is heavily exacerbated by the rise of Private Equity (PE) in dentistry. Through the acquisition of independent practices by Dental Support Organizations (DSOs), PE firms have centralized operations to prioritize profit-driven growth and eventual buyouts. Ethically, this paradigm risks subordinating the American Dental Association principles of patient welfare and clinical autonomy to short-term investor yields (15). When financial metrics dictate care, clinicians are often pressured into ethical compromises, such as overtreatment or prioritizing lucrative elective procedures over the comprehensive, non-discriminatory care that medically vulnerable patients desperately require (16, 17).

Research on what motivates dental students to pursue SCD practice underscores the relationship between structured preparation and workforce pipeline. Preparedness to care for disabled patients was positively associated with intent to serve special care populations, suggesting that competency development itself shapes the workforce (18). How programs are designed and resourced carries direct consequences for who ultimately serves these populations.

A competency-based approach to SCD has the benefit to articulate clear expectations for medical risk assessment, disability-informed care, interprofessional collaboration, ethical decision-making, and continuity of care. Competencies should be observable, assessable, and transferable across settings, enabling greater consistency in training outcomes and workforce preparedness. SCD educators who align curriculum with competency-based medical education and interprofessional learning frameworks strengthen the credibility of dental training with multidisciplinary care teams (19) and proponents should align education with broader health professions education trends such as competency-based medical education and interprofessional learning to enhance credibility of medical-dental integration with multidisciplinary care teams. Competency development for SCD extends across the full dental team. Dental hygienists and assistants who provide much of the preventive and maintenance care in community and long-term care settings require preparation for medical complexity and interprofessional communication that AEGD and GPR training environments are particularly well positioned to model.

The educational value of these advanced dental training extends beyond dental residents. Many of the training settings provide interprofessional proximity and shared patient encounters that medical, nursing, pharmacy, and allied health students rarely access in dental school contexts. Medical schools have long struggled to integrate meaningful oral health content into their curricula, with more than 90 percent of U.S. medical school graduates in 2012 reporting they were not well trained to address oral health topics, and two-thirds of osteopathic medical schools offering fewer than six hours of oral health training as recently as 2018 (20). The structural barriers to integration in these settings, including lack of time, lack of dental expertise on medical faculty, and absence of institutional prioritization, are well documented (20). Advanced dentistry programs, embedded within the same institutional environments as medicine and nursing trainees, offer a solution to this problem through direct clinical encounter rather than formal curricular add-on. The AEGD and GPR resident who consults on an in-patient floor, presents at a team huddle, or participates in a multidisciplinary case conference is delivering oral health education to medical colleagues in the context Gill and colleagues identify as most effective: clinically grounded, bidirectional, and patient-centered (20, 21).

A systems approach to health professions education must also attend to faculty development, accreditation, and institutional incentives. Without recognized standards, programs providing meaningful medical-dental integration will remain isolated examples rather than components of a broader global movement (22). Articulating shared competencies and educational frameworks facilitates curriculum development, program evaluation, and clearer professional pathways. Few dental training environments have accumulated the clinical depth and interprofessional experience that these advanced dental residency programs bring to competency development for complex patient care (23, 24).

## Clinical pathways and standards of care

The private practice model that dominates oral health delivery in the United States is structurally ill-suited to the needs of patients served by SCD. A 2024 national cross-sectional analysis identified geographic clustering of dental shortage areas corresponding closely with rural location, racial segregation, and high area deprivation, conditions that describe the communities where medically complex, disabled, and frail patients are disproportionately concentrated (1). Distance and geography compound these disparities, creating access barriers that accumulate for patients managing disability or chronic illness alongside transportation limitations (3). The fee for service structure that dominates private practice dentistry creates economic incentives poorly suited to the care of medically complex populations; community health centers serving Medicaid patients operate under different reimbursement frameworks, but chronically low Medicaid rates and limited private dental insurance coverage leave providers financially constrained regardless of setting (4, 25).

Hospital-based practice and integrated care models offer structural advantages that align with the demands of SCD. Within the hospital or integrated care system, oral health care can be coordinated with medical treatment, rehabilitation services, nursing care, and social support enabling a more comprehensive response to complex patient care needs. These environments encourage shared medical records access, interdisciplinary consultation, and standardized safety protocols essential for risk management in SCD populations. A cross-sectional audit of two large Australian hospital-based special needs dental referral centers demonstrated the breadth of patient complexity referred for specialist care, with complex medical patients and patients with disabilities or psychiatric conditions well represented, and with general dentists consistently identifying this population as requiring hospital-level specialist management (26).

The integration of oral care into broader clinical pathways is a patient safety matter. Oral health remains largely disconnected from geriatric hospital services, creating a pattern of delayed diagnosis, reactive treatment, and preventable decline in quality of life for exactly the populations SCD is designed to serve (27). Patient safety and quality improvement frameworks, well established in medicine, have only recently begun to be applied systematically to oral health care delivery, including within the management of musculoskeletal orofacial pain, where principles of risk assessment, care coordination, and institutional accountability are essential to safe and effective outcomes (28). The same absence of standardized oral health protocols has been documented in hospital stroke units internationally: a 2025 scoping review found that while education improved knowledge and attitudes among nursing and allied health staff, no evidence linked these educational efforts to actual changes in clinical practice or patient outcomes, confirming that knowledge transfer alone is insufficient without structural integration of dental expertise into hospital care teams (29). Across these contexts, the pattern is consistent: when oral health care is treated as a peripheral accommodation rather than an accountable clinical function embedded in institutional systems, patient safety suffers.

Community-based, mobile, and long-term care facility models also play a critical role in extending SCD reach, and school-based programs contribute meaningfully to oral health access for pediatric populations, including children with disabilities and special health care needs (30). When aligned with expertise in advanced dental residency programs, and supported by digital communication tools, these models can improve continuity of care, reduce fragmentation, and strengthen patient safety across care settings. Their effectiveness depends on clearly defined roles, referral pathways, and communication channels that isolated models of care frequently lack (31). Furthermore, the integration of Artificial Intelligence (AI) in dentistry significantly enhances these connected networks. AI-driven screening and teledentistry tools enable remote image analysis and automated triaging, flagging declining oral health early and reducing the burden on human specialists (32). Caregiver education in oral hygiene and preventive care is an equally critical component of SCD delivery, particularly for patients in long-term care and home-based settings where consistent oral hygiene depends on trained caregivers who understand its clinical significance for medically complex individuals. To support this, AI-powered applications can provide caregivers with 24/7 personalized oral hygiene guidance, interactive training, and risk-assessment tracking tailored to individual patient needs (33).

The barriers to oral health integration in primary and community care settings have been systematically mapped. A scoping review of 58 publications identified discipline-oriented education, absence of political leadership, and fragmented service organization as the dominant structural barriers, while supportive policies, interdisciplinary education, and geographic proximity of dental and medical services were the primary facilitators (34). Care delivery reform and educational reform are interdependent; neither succeeds without the other.

An interprofessional experience pairing dental hygiene students with family medicine residents in Medicaid Managed Care settings demonstrated that family medicine providers reported high value from oral health training, including improved recognition of oral conditions they would otherwise have missed, with direct implications for medically complex populations who rely on primary care as their primary healthcare touchpoint (35). Veterans represent one such population: data from the National Health and Nutrition Examination Survey confirm that veterans carry higher odds of overall dental disease than non-veterans, with depression compounding this burden further, while the majority of veterans lack Veterans Health Administration dental benefits and face substantial financial barriers to care (36). Among veterans with spinal cord injuries, 63 percent lacked VA dental care eligibility and financial barriers remained the dominant obstacle to access regardless of injury level or age (37). The VA system has long been a training ground for advanced general dentistry programs serving populations whose complexity exceeds what private practice can manage, including patients whose histories of combat trauma, traumatic brain injury, and military sexual trauma require care delivered within trauma-informed frameworks that conventional dental training rarely address (36, 37).

## Policy, recognition, and workforce structures

Educational reform and clinical innovation require policy frameworks that recognize and support the delivery of complex oral care within health systems. Current funding mechanisms, workforce planning models, and regulatory structures are poorly aligned with patient needs. They fail to account for the additional time, coordination, and interprofessional engagement required to care for individuals with complex medical needs, disabilities, and frailty.

Formal specialty recognition carries significant implications for legitimacy, funding, and workforce development. Special care dentistry illustrates how recognition through mechanisms including CODA accreditation, graduate medical education funding, and institutional clinical accountability can establish a sustainable infrastructure for complex care. Policy frameworks that acknowledge SCD as an essential health system capacity rather than a niche specialty may offer more flexible and context-responsive pathways for recognition and support.

Reimbursement structures present a parallel challenge. Payment models prioritizing procedural volume over care coordination do not reflect the resource demands of SCD practice. This misalignment discourages clinicians from engaging with high-need populations and undermines continuity of care. Aligning payment structures with value-based care principles, including risk adjustment and support for interdisciplinary collaboration, would better reflect the realities of SCD work, and is well suited to be in integrated care settings where oral health services interface with broader institutional priorities.

A 2021 analysis of oral health's role in global health identified fundamental gaps in the evidence base, including in epidemiology and health information systems, harmonization of evidence for prevention, equity, and treatment, and strategies for delivering essential quality care without causing financial hardship (38). The authors argued that oral health must be repositioned from a professional concern to a population health priority, a reframing with direct relevance to SCD. The field has produced important clinical work but has not generated the structured research agenda or the policy advocacy infrastructure needed for systems-level influence. A mapping review of oral health interventions for people with diabetes found that policymaker-level evidence is critically thin, representing a single study among 76 reviews included, and identified integration of periodontal care into chronic disease management pathways as a top future research priority (39). The absence of a comparable research agenda for SCD is itself a gap that SCDA is positioned to address.

## Technological innovation in special care dentistry

Technology has the potential to transform care delivery in complex care settings where systems-based thinking is critical. For populations characterized by medical complexity, disability, and frailty, inconsistent assessment processes, fragmented communication, and poor care coordination represent significant barriers to quality care and patient safety. Digital and automated tools can reduce the variability by implementing standardized processes, improving continuity of care across settings, and supporting interdisciplinary collaboration as central objectives of medical-dental integration.

Applications relevant to SCD include structured patient assessment and risk stratification tools, digital intake platforms, standardized medical-dental history instruments, and clinical decision support systems that assist providers in identifying medical risks, functional limitations, and care dependencies. These tools could support more consistent treatment planning, reduce reliance on informal clinical judgement, and facilitate appropriate referrals or escalation of care. In hospital and integrated care environments, they could improve alignment with medical records and institutional safety protocols.

Tele-dentistry extends the reach of medical-dental integration within complex health systems. For individuals with mobility limitations, cognitive impairment, or residence in long-term care facilities, virtual consultations can support triage, follow-up, caregiver engagement, and interdisciplinary case discussion. The value of tele-dentistry lies in extending and supplementing continuity rather than replacing clinical care, a distinction that matters considerably for populations whose access to in-person services is structurally limited (6).

Digital workflow and automation tools offer opportunities to address administrative and logistical challenges that may disproportionately impact SCD practice. Scheduling systems that accommodate extended appointment times, care coordination platforms that facilitate cross-disciplinary communication, and documentation support that improves clinical efficiency while reducing provider burden all serve populations whose care demands exceed what conventional dental workflows were designed to handle. These tools must be designed to function within integrated systems where oral health aligns with institutional workflows and regulatory requirements.

Adoption of technology in health care requires attention to ethical and equity considerations. Digital tools, artificial intelligence and automated systems risk exacerbating existing disparities when they rely on datasets that underrepresent medically complex, disabled, or frail populations. Accessibility, transparency, and bias mitigation must be foundational design principles. Trust, communication and individualized care are vital to effective SCD practice, and these values must be embedded within educational frameworks, standardized clinical pathways, and supportive policy environments. Hospital dentistry and integrated care settings are practical sites for evaluating how digital and automated tools can enhance safety, coordination, and equity in complex care.

## Limitations/challenges

For all its structural advantages, the hospital dentistry model presents with real and persistent barriers. From the provider perspective, AEGD and GPR programs remain chronically underfunded, with stipends unable to compete with private practice salaries/income, faculty vacancies that compromise training quality, and institutional administrators who view hospital dental programs as peripheral to the hospital's core mission. Patients face a different set of obstacles from transportation and mobility limitations, long waits for hospital-based specialist appointments, and difficulty in prioritizing dental care with multiple competing medical demands. For those who require general anesthesia, access to operating room time is a constraint. Operating room (OR) scheduling prioritizes life-saving surgical procedures with dental cases routinely deferred and waiting lists that extend months for patients with severe disabilities and no viable outpatient alternative (40).

The reimbursement environment further compounds these challenges. For patients who require sedation and can be managed on the outpatient setting, dental anesthesiologists are often hesitant to accept Medicaid and Medicare reimbursement due to low payment rates and complex billing processes. Medicare also does not cover routine dental care, leaving a structural gap for elderly and medically complex patient populations that SCD is designed to serve. At age 65, low-income patients who become dual-eligible for Medicare and Medicaid gain no federal dental benefit through Medicare, while their Medicaid dental coverage remains subject to wide variations in scope by state, reimbursement rates, and administrative requirements. In many jurisdictions, reimbursement rates are too low for sustainable provider participation, and procedural coverage gaps leave medically necessary treatment unfunded even when patients present for care (25).

The shortage of dentists trained to manage medically complex patients in hospital settings means that clinical capacity is insufficient to meet demand even where institutional infrastructure exists. These barriers reinforce the case for sustained investment in training, reimbursement reform, and workforce development.

## Call to action: advancing care

Transformation requires a shift from fragmented, accommodation-based practice toward coordinated, system-level care that treats complexity as a defining feature rather than an exception. Special Care dentistry provides a practical foundation for this transformation. A systems approach to education, standardized clinical pathways, responsible technological innovation, and supportive policy frameworks can help SCD evolve into a more coherent discipline capable of meeting 21st-century population health needs.

This Research Topic, *Transforming Special Care Dentistry: Frameworks for Education, Clinical Excellence, and Technological Innovation*, offers a timely opportunity. The field needs researchers to develop evidence for competency-based education and evaluate integrated care models; educators and clinical leaders to define competencies that reflect real-world complexity; and policymakers to fund pathways for the care of vulnerable populations. These tasks are interdependent and cannot be accomplished in silos.

Advancing SCD is a health system responsibility, not a professional identity exercise. Placing it within broader frameworks of quality, safety, and equity, informed by the lessons of integrated, hospital-based care, can move oral health for complex populations from the margins of the profession to the center of oral health policy and practice. This article offers an organizing framework for that transition and invites the interprofessional engagement needed to carry it forward.

## Data Availability

The original contributions presented in the study are included in the article/Supplementary Material, further inquiries can be directed to the corresponding authors.
