# Supplementary figures and images for: From hospital dentistry to health systems: advancing special care dentistry through education, integration, and technology
Source: Front Oral Health. 2026 Jul 17;7:1910418. doi: 10.3389/froh.2026.1910418 (PMC13424287; doi:10.3389/froh.2026.1910418)

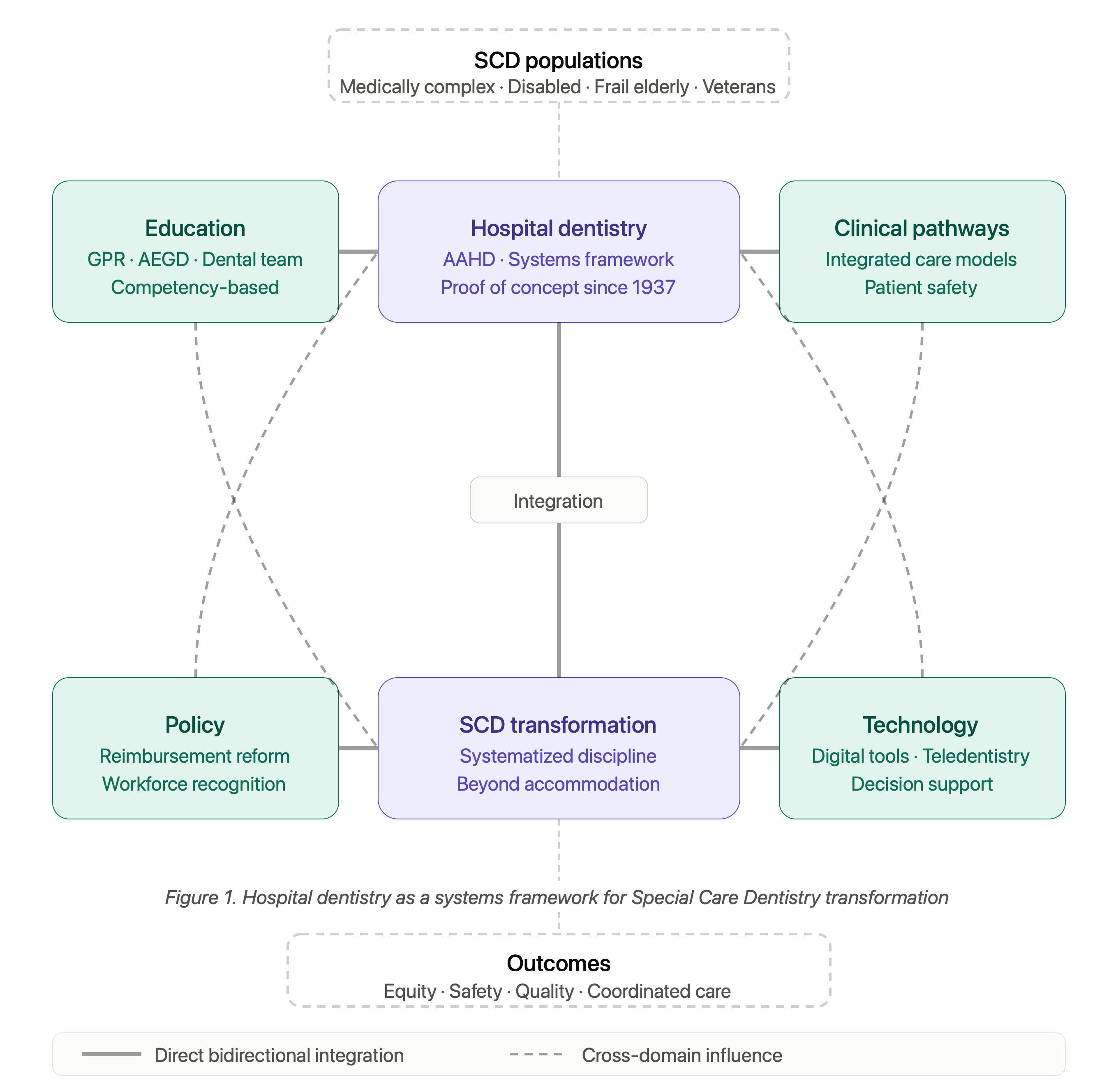

Supplement: Supplementary file 1 [file Image1.png]
